# NhaA Na^+^/H^+^ Antiporter Mutants That Hardly React to the Membrane Potential

**DOI:** 10.1371/journal.pone.0093200

**Published:** 2014-04-03

**Authors:** Dudu Alkoby, Abraham Rimon, Maral Burdak, Miyer Patino-Ruiz, Octavian Călinescu, Klaus Fendler, Etana Padan

**Affiliations:** 1 Department of Biological Chemistry, Alexander Silberman Institute of Life Sciences, Hebrew University, Jerusalem, Israel; 2 Department of Biophysical Chemistry, Max-Planck Institute for Biophysics, Frankfurt/Main, Germany; University of Cambridge, United Kingdom

## Abstract

pH and Na^+^ homeostasis in all cells requires Na^+^/H^+^ antiporters. The crystal structure, obtained at pH 4, of NhaA, the main antiporter of *Escherichia coli*, has provided general insights into an antiporter mechanism and its unique pH regulation. Here, we describe a general method to select various NhaA mutants from a library of randomly mutagenized NhaA. The selected mutants, A167P and F267C are described in detail. Both mutants are expressed in *Escherichia coli* EP432 cells at 70–95% of the wild type but grow on selective medium only at neutral pH, A167P on Li^+^ (0.1 M) and F267C on Na^+^ (0.6 M). Surprising for an electrogenic secondary transporter, and opposed to wild type NhaA, the rates of A167P and F267C are almost indifferent to membrane potential. Detailed kinetic analysis reveals that in both mutants the rate limiting step of the cation exchange cycle is changed from an electrogenic to an electroneutral reaction.

## Introduction

Living cells are critically dependent on processes that regulate intracellular pH, Na^+^ content and volume [1]. Na^+^/H^+^ antiporters play a primary role in these homeostatic mechanisms (recently reviewed in [2] and [3]). They are found in the cytoplasmic and intracellular membranes of most organisms from bacteria to humans and they have long been human drug targets [4].

NhaA, the principal Na^+^/H^+^ antiporter in *Escherichia coli*, is indispensable for adapting to high salinity, challenging Li^+^ toxicity, and growing at alkaline pH (in the presence of Na^+^ [5]). It is widely spread in enterobacteria [6] and has orthologs throughout the biological kingdoms, including humans [7].

Several biochemical characteristics of NhaA underpin its physiological roles: very high turnover (10^5^ min^−1^) [8], electrogenicity with a stoichiometry of 2H^+^/Na^+^ [9] and strong pH dependence [8], a property it shares with other prokaryotic [5], as well as eukaryotic Na^+^/H^+^ antiporters (reviewed in [10], [11], [12], [13]).

In a series of reports [14], [15], [16], we have studied the kinetics and partial reactions of the NhaA transport cycle using SSM (solid supported membrane)-based electrophysiology. Forward and reverse transport directions were investigated using preparations of inside-out and right-side out oriented transporters with Na^+^ or H^+^ gradients as the driving force [15]. This work showed that NhaA is a symmetric transporter and advanced a kinetic model of the NhaA transport cycle. The model is based on the ‘alternate accessibility’ mechanism in which a single binding site is alternating across the membrane [17], [18]. H^+^ and Na^+^ were shown to compete for a single binding site of NhaA and such a competition explains many phenomena of NhaA pH regulation [15].

**Figure 1:**
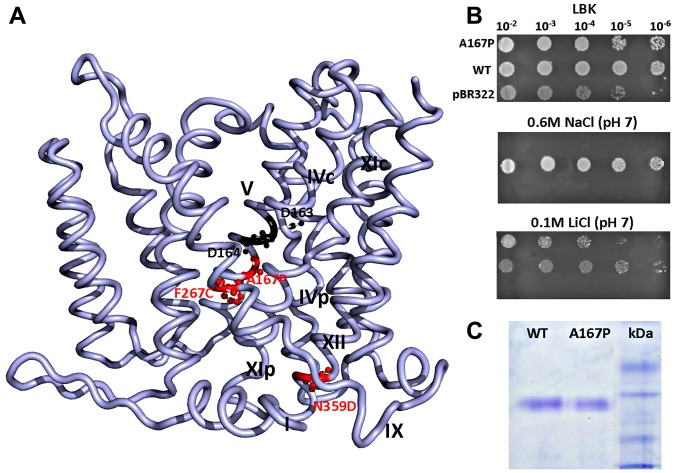
A167P NhaA mutant growth and expression. (A) The double mutant A167P (TM V)-N359D (TM XII) and the previously [24] isolated mutant F267C (TM IX) are shown in red (ball and stick) on the NhaA structure without TM I, for clarity (ribbon presentation by PyMol). Asp163 and Asp164 are shown in black at the putative active site. (B) Growth of *E. coli* EP432/A167P on agar plates of LBK (top panel) and on selective media: high Na^+^ (middle panel) and high Li^+^ (lower panel) at pH 7. (C) Expression level of A167P protein in isolated membrane vesicles from *E. coli* EP432/A167P as compared to the WT was determined as described in Materials and Methods.

The crystal structure of NhaA crystallized at acidic pH [19] has provided the first structural insights into the antiport mechanism and pH regulation of a Na^+^/H^+^ antiporter [20]. NhaA consists of 12 transmembrane helices (N and C termini on the cytoplasmic side of the membrane) organized in a new fold; TMs (Trans Membrane segments) III, IV and V are topologically inverted with respect to TMs X, XI and XII. In each repeat, one TM (IV and XI, respectively) is interrupted by an extended chain crossing each other in the middle of the membrane. As a result, two short helices (IVc, IVp, and XIc, XIp, respectively) are left oriented to the cytoplasm (c) or periplasm (p) ([19] and Fig. 1A). This non-canonical TM assembly creates a delicately balanced electrostatic environment in the middle of the membrane at the ion binding site(s), which likely plays a critical role in the cation exchange activity of the antiporter.

Remarkably, many of the structural folds of secondary transporters deciphered since NhaA also include inverted topological repeats containing interrupted helices with functional implications similar to those for NhaA (reviewed in [2], [3], [21], [22]). Yet, there are at least three different folds among secondary transporters as exemplified by LacY, LeuT and NhaA [23]. However, the transporters sharing a fold do not share sequence similarity.

The NhaA structure allowed the interpretation of mutational data in a rational way [20]. Projecting the mutants location on the structure revealed two functional regions: (a) a cluster of amino acyl side chains, about 9 Å away from the catalytic site, that modulate the response to pH (residues on loops VIII–IX, TMs IX, IVc, II), and (b) a catalytic region containing the ion-binding sites (residues on TMs IV and V). This suggests that in addition to regions involved in substrate transport, residues remote from the active site participate in the substrate response of NhaA. Electrophysiological analysis supported this contention [16].

Nevertheless, the crystal structure is a single snap-shot; determined at pH 4 when NhaA is down-regulated [19] while NhaA is activated at pH 6.5 and reaches maximal activity at pH 8.5 [8]. Therefore, in addition to attempting to crystallize an active conformation of NhaA, we study its functional dynamics both *in vitro* and *in vivo* at physiological pH. In this respect, NhaA mutants that alter the antiport activity are of special interest for identifying the residues that are most likely to contribute to the ion translocation machinery and its regulation at physiological pH. Here we describe a selection method for isolation of mutants impaired in various functional properties of NhaA with mutant A167P, as an example (Fig. 1A). Remarkably, this mutant, which is located in TM V in proximity to the cation binding site, showed a growth and activity phenotype [16] very different from the WT (wild type) and very similar to a previously isolated [24] mutant F267C in TM IX (Fig. 1A). In particular, these two mutants did not show the canonical response to a change in the membrane potential characteristic of an electrogenic antiporter such as WT NhaA; turnover of an electrogenic antiporter creates a membrane potential which slows its rate. Therefore, the rate of a ΔpH driven ^22^Na^+^ uptake into NhaA proteoliposomes increases drastically upon collapse of the membrane potential [3].

In marked contrast, the rates of mutants A167P and F267C hardly change under similar experimental setup. Such a behavior is characteristic of electroneutral transporters which do not produce membrane potential during turnover. Here we show that a reduced turnover rate and/or a switch of the rate limiting step of the transport cycle from an electrogenic to an electroneutral step can yield a phenotype of an electroneutral transporter.

## Results

We describe a method to select for various NhaA mutants and the isolation and characterization of a mutant, A167P, in TM V, in proximity to the cation binding site [25]. A preliminary electrophysiological characterization of this variant has been published elsewhere [16]. Similar to F267C, a previously isolated mutant in TM IX [24], A167P does not show the canonical response of an electrogenic transporter like NhaA to a change in membrane potential. Here we study these mutants and reveal that a change in the mutant’s transport cycle, from an electrogenic rate limiting step to an electroneutral step is the most likely reason for the mutant’s cryptic phenotype.

### Isolation of NhaA Mutants

Following PCR-based random mutagenesis, the mutagenized NhaA-encoding plasmid (pAXH3) was transformed into KNabc, an *E. coli* strain in which the genes encoding the specific Na^+^/H^+^ antiporters, NhaA and NhaB, and the nonspecific ChaA had been inactivated [26]. The latter antiporter antiports K^+^ in addition to Na^+^ and Li^+^, and its ability to antiport excess K^+^ is essential for cell adaptation to high K^+^. The transformants were first grown on non-selective medium LBK at pH 7 to form colonies, producing a cell library with mutated plasmidic NhaA. These colonies were then replica-plated on LBK-based selective media: Na^+^ (0.6 M) or Li^+^ (0.1 M) at pH 7 and pH 8.2. These selective media were expected to identify various potential NhaA mutants on the basis of the growth phenotypes as follows: 1) Mutants that cannot grow on any of the selective media are impaired in expression and/or NhaA antiporter activity [5]. 2) Mutants that grow on only one of the selective media at both pH 7 and pH 8.2 are impaired in ion selectivity (transporting either Na^+^ or Li^+^). 3) Mutants that grow on the selective media at pH 7 but not at pH 8.2 are impaired in pH response and/or energy coupling because both alkaline pH activation of NhaA and its stoichiometry of 2H^+^/Na^+^ (electrogenicity) are critical for *E. coli* growth at alkaline pH in the presence of Na^+^/Li^+^ [5].

Among 60,000 screened cells, 10 mutants grew only on the non-selective media, and nine carrying two or more mutations in *nha*A grew on certain selective media, verifying the efficiency of mutagenesis. One variant with double mutations, KNabc/A167P/N359D (Fig. 1A) showed a more complex than expected growth phenotype; it grew on the non-selective media (LBK) similar to the WT (data not shown), but as opposed to the WT that grew on both selective media at pH 7 and pH 8.2, the mutant grew slowly on the Li^+^ selective medium at pH 7 but not at pH 8.2 and did not grow at all on Na^+^ (0.6 M) selective media neither at neutral pH nor at alkaline pH (Table 1). Therefore, we studied it further. First we constructed NhaA carrying only one of the two mutations and found out that mutation A167P is responsible for the phenotype and even grows better than the double mutant on the Li^+^ selective medium at neutral pH (Table 1). Remarkably, A167P is located on TM V in proximity to the NhaA active site (Asp163 and Asp164, [25], Fig. 1A).

**Table 1:** Expression level, growth phenotype and Na^+^/H^+^ antiporter activity of variant A167P.

Variants^a^	Expression^b^	Growth Phenotype^c^				Apparent *K*_m_ d		Activity^e^ %^f^	
	(% of WT)	0.6 M NaCl		0.1 M LiCl		pH 8.5, mM		pH 8.5	
		pH:		pH:		Na^+^	Li^+^	Na^+^	Li^+^
		7	8.2	7	8.2				
A167P/N359D	ND	+/−	−	+	−	5.34	0.81	78	72
A167P	ND	−	−	++	−	1.62	0.17	86	72
WT	ND	+++	+++	+++	++	ND	ND	90	83
pBR322	–	−	−	−	−	ND	ND	0	0
A167P/N359D	ND	−	−	++	−	ND	ND	ND	ND
A167P	70	−	−	+++	−	1.2	0.4	55	48
WT	100	+++	+++	+++	++	0.16	0.03	98	91
pBR322	–	−	−	−	−	ND	ND	0	0
F267C^g^	95	+++	−	ND	ND	0.8	ND	90	ND

a For characterization of variant A167P, the *E coli* KNabc (lines 1–4) and *E coli* EP432 (lines 5–9) cells were transformed with plasmids expressing the indicated variants. The positive and negative controls were cells transformed with pAXH3 expressing WT- NhaA and pBR322-the empty vector, respectively.

b Expression level in the membrane is expressed as percentage of control cells (WT).

c Growth experiments were conducted at 37°C on LB modified agar plates containing 0.6 M NaCl at pH 7 or pH 8.2 or 0.1 M LiCl at pH 7 or pH 8.2; +++, number and size of the colonies after 48 h of incubation of the control; ++, same number of colonies as the control but smaller in size; +, both size and number of colonies reduced compared to controls; -, no growth.

d The apparent *K*_m_ for the ions was determined at pH 8.5, as described in “Materials and Methods”.

e Na^+^/H^+^ and Li^+^/H^+^ antiporter activity in everted membrane vesicles at pH 8.5 was determined with 10 mM NaCl or LiCl.

f Activity is expressed as percentage of dequenching. ^g^The respective values for variant F267C were taken from [24].

ND, not determined.

### Mutant A167P, Expression in the Membrane and Growth Phenotype

To characterize mutant A167P with respect to expression, growth and antiporter activity (Table 1), the mutated plasmid was transformed into EP432 [27], an *E. coli* strain that lacks the two Na^+^-specific antiporters (NhaA and NhaB). Similar to *E. coli* KNabc, this strain does not grow on the selective media nor does it exhibit Na^+^/H^+^ antiporter activity in isolated everted membrane vesicles, unless transformed with a plasmid encoding an active antiporter (reviewed in [6] and Table 1).

Mutant A167P was significantly (≥70%) expressed in EP432 cells as compared to the expression level of the WT (100%, Fig. 1C and Table 1). Notably, because A167P was obtained in plasmid pAXH3, a multi-copy plasmid, the level of its expression was far above the level expressed from a single chromosomal gene which confers a Na^+^-resistance phenotype [28]. The growth phenotype of *E. coli* EP432/A167P was similar to that of *E. coli* KNabc/A167P (Table 1). It grew on the Li^+^ selective medium at pH 7 but not at pH 8.2 and did not grow on the Na^+^ selective media at either pH 7 or at pH 8.2 (Table 1, Fig. 1B).

### The Na^+^/H^+^ Antiporter Activity Assessed in Everted Membrane Vesicles

**Figure 2:**
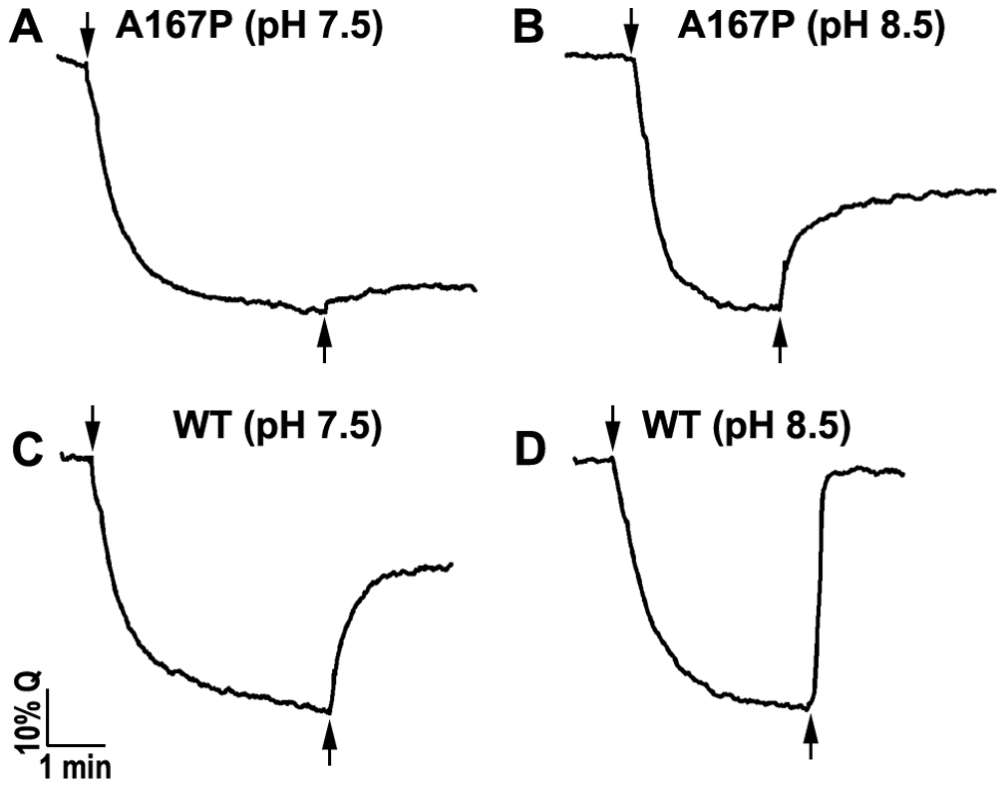
Na^+^/H^+^ antiporter activity of variant A167P in isolated everted membrane vesicles. For measurement of the Na^+^/H^+^ antiporter activity at the indicated pHs, *E. coli* EP432/A167P cells expressing variant A167P (A and B) or the wild type (C and D) were grown in LBK (pH 7.0) and everted membrane vesicles were isolated. The ΔpH across the membranes was determined using acridine orange, a fluorescence probe of ΔpH. The reaction mixture (2.5 ml) contained 50–100 μg of membrane protein, 0.5 μM acridine orange, 150 mM KCl, 50 mM BTP buffer, 5 mM MgCl_2_ and the pH was titrated with HCl. The data of typical experiments are shown. At the onset of the reaction, D-lactate (2 mM) was added (downward facing arrow) and the fluorescence quenching (*Q*) was recorded until a steady-state level of ΔpH (100% quenching) was reached. NaCl (10 mM) was then added (upward facing arrow), and the new steady state of fluorescence obtained (dequenching) was monitored. Fluorescence dequenching indicated that protons are exiting the vesicles in response to Na^+^ influx via the antiporter. All experiments were repeated at least three times with practically identical results.

The Na^+^/H^+^ and Li^+^/H^+^ antiport activities of A167P were measured in everted membrane vesicles isolated from *E. coli* EP432/A167P (Table 1, Fig. 2A, B) and *E. coli* KNabc/A167P cells (Table 1). Cells transformed with plasmid pAXH3 encoding WT NhaA or the vector plasmid, pBR322, served as positive and negative controls, respectively (Fig. 2C, D and Table 1). The activity was estimated from the change caused by either Na^+^ (Fig. 2, Table 1) or Li^+^ (Table 1) to the ΔpH maintained across the membrane, as measured by acridine orange, a fluorescent probe of ΔpH. After energization (Fig. 2*A*, *down facing arrow*) with D-lactate, quenching of the fluorescence achieved a steady state level and then Na^+^ (Fig. 2*A*, *up facing arrow*) or Li^+^ (data not shown) was added. Dequenching of the fluorescence indicates that protons are exiting the vesicles in exchange for Na^+^ or Li^+^ (further details in Materials and Methods). The extent of activity (maximal dequenching), at pH 8.5, was determined for each treatment (Table 1 and data not shown). *E. coli* EP432/A167P was inactive with Na^+^ or Li^+^ at pH 7.5 but, at pH 8.5, it was about 50% active as compare to the WT. Somewhat higher values were obtained with *E. coli* KNabc/A167P (Table 1).

The apparent *K*_m_ values for Na^+^ and Li^+^ of membrane vesicles isolated from *E. coli* EP432/A167P were about 10 fold higher than that of the WT at pH 8.5 (1.2 mM and 0.4 mM versus 0.16 and 0.03 respectively) (Table 1). The results obtained in *E. coli* KNabc/A167P were similar to those of *E. coli* EP432/A167P (Table 1).

### The pH Dependence of the Na^+^/H^+^ Antiporter Activity of EP432/A167P in Everted Membrane Vesicles

**Figure 3:**
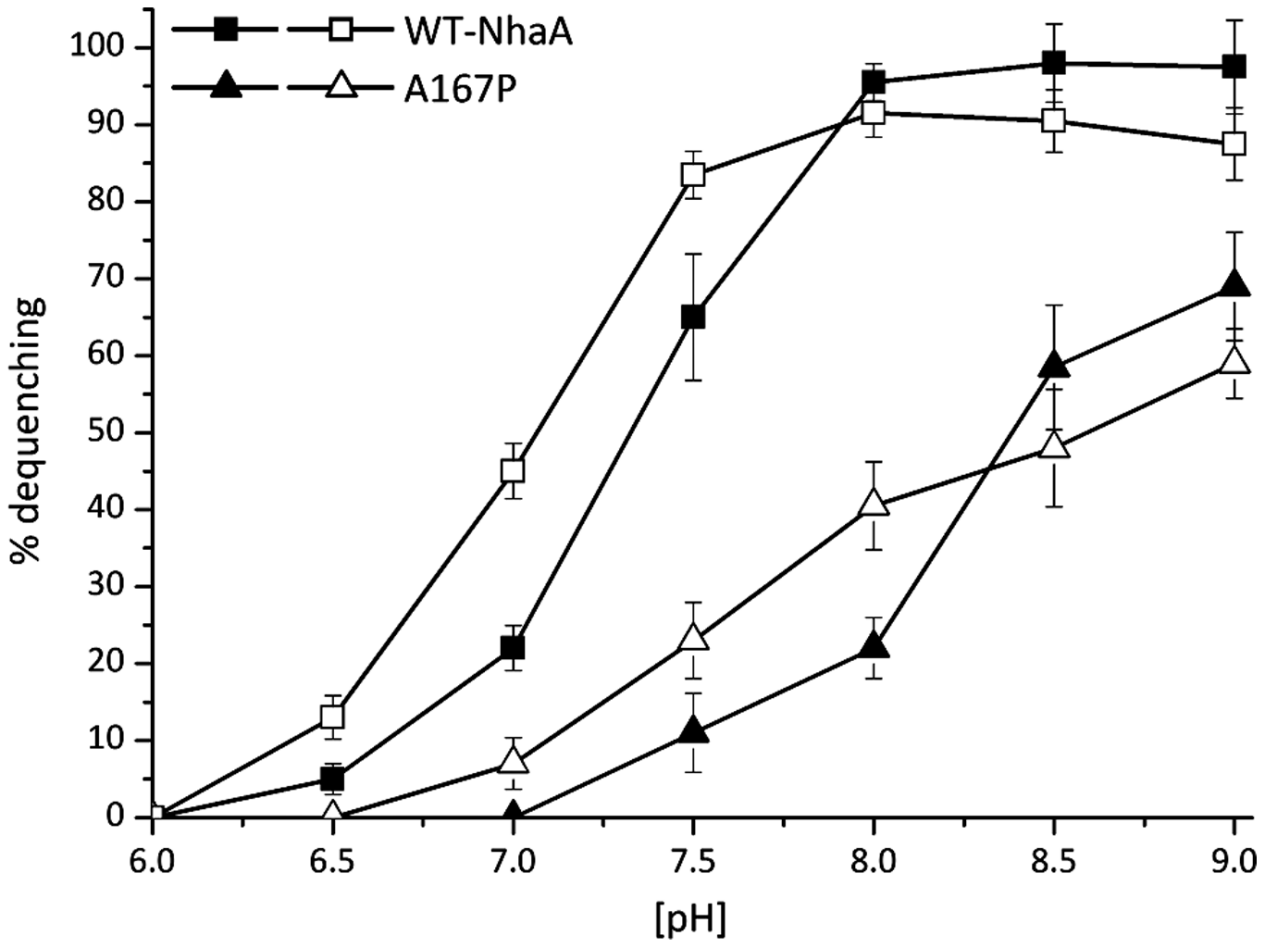
pH dependence of the Na^+^, Li^+^/H^+^ antiporter activity in everted membrane vesicles of variant A167P. Everted membrane vesicles were prepared from EP432 cells grown in LBK (pH 7) and expressing WT (**□**) or A167P (Δ). The Na^+^/H^+^ and Li^+^/H^+^ antiporter activity was determined in the presence of 10 mM NaCl (filled symbols) or 10 mM LiCl (open symbols) at the indicated pH values, using acridine orange fluorescence to monitor ΔpH. Results are expressed in % of maximal dequenching of the fluorescence due to cation addition. All experiments were repeated at least three times with nearly identical results.

We have previously shown [29], [30] that certain mutations in NhaA only affect the apparent *K*_m_, and not the pH dependence of the exchanger. These mutants show an altered pH dependence at non-saturating Na^+^ concentrations but a pH dependence similar to the wild type at saturating Na^+^. By contrast, other mutations retain an altered pH dependence at both saturating and non-saturating Na^+^ concentrations, irrespective of whether the Na^+^ affinity is changed or not from the WT. Therefore, the pH profile of the antiporter activity in membrane vesicles isolated from *E. coli* EP432/A167P was measured at saturating- concentrations of the cations (Fig. 3). The pH dependence of both Na^+^/H^+^ and Li^+^/H^+^ antiport activity of the variants was shifted by about 1 pH unit to the alkaline side as compared to the WT.

### The Rate of Variant A167P is Hardly Affected by a change in the Membrane Potential

**Figure 4:**
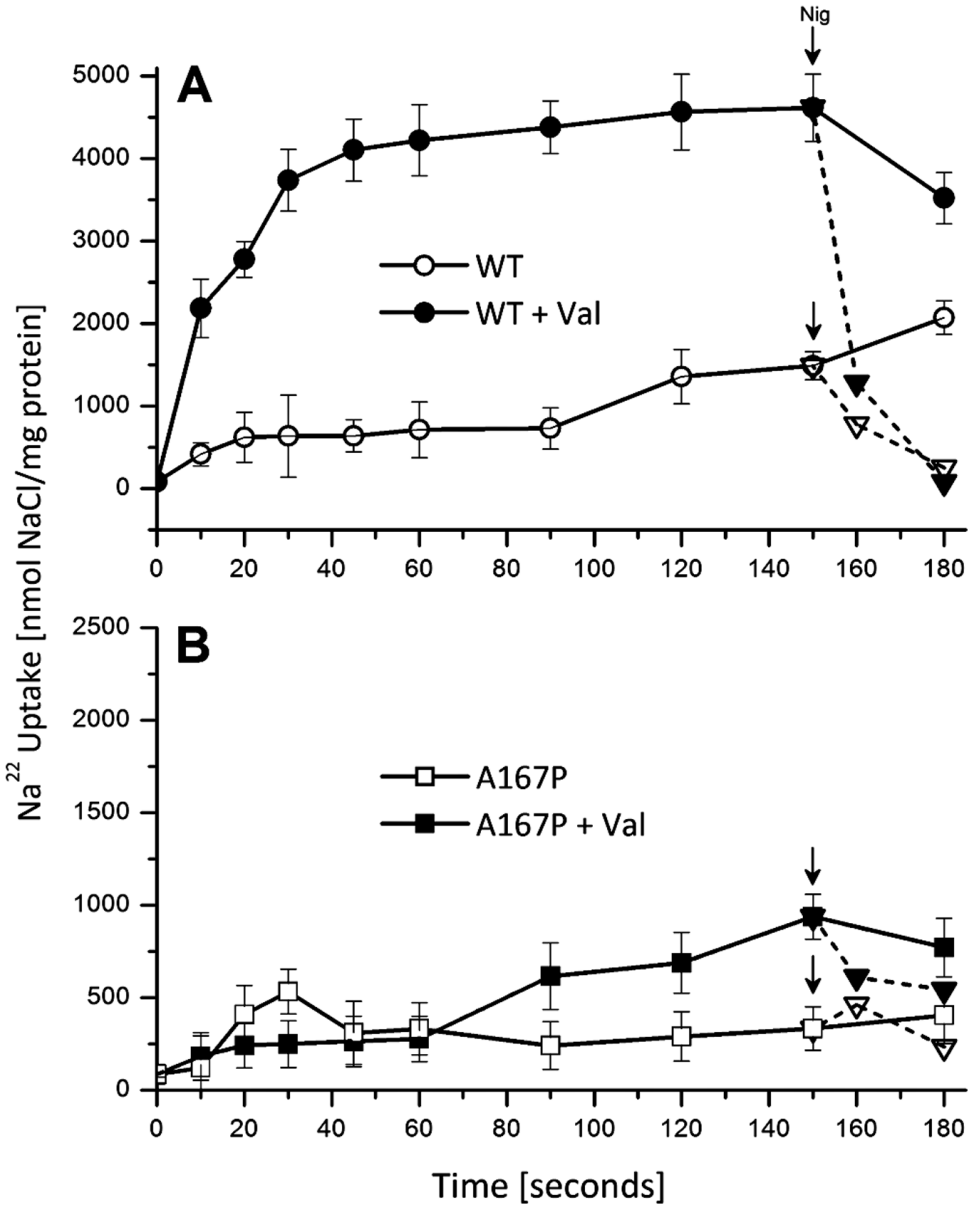
The effect of a counter-ion on ^22^Na transport by A167P proteoliposomes. The affinity purified proteins of WT (A) and A167P (B) proteins were reconstituted into proteoliposomes and ΔpH (acidic inside)-driven ^22^Na uptake was determined as described under “Experimental Procedures”. The reaction mixture (500 μl) contained 150 mM choline chloride, 10 mM Tris/Hepes (pH 8.6), 2 mM MgS0_4_, 10 mM KCl and 50 μM ^22^NaCl (1 μC/ml). Where indicated (filled symbols), valinomycin (5 μM) was added from zero-time. At steady state nigericin (1 μM) was added to the various reaction mixtures (dashed line).

WT NhaA is electrogenic because of its 1Na^+^/2H^+^ stoichiometry [9]. We have previously demonstrated that NhaA as any other electrogenic secondary transporter, is very sensitive to the presence of membrane potential, either imposed or produced during its turnover. Thus, when NhaA is reconstituted into proteoliposomes and its ΔpH-driven ^22^Na uptake activity is determined in a reaction mixture devoid of any permeable ion, a slow rate and a low steady state is reached within one minute because of the ΔΨ it produces ([9] and Fig. 4A). In marked contrast, in the presence of a permeant ion (valinomycin in the presence of K^+^) both the uptake rate and the steady state of the antiporter activity drastically increase around 4–5 fold ([9], review in [1] and Fig. 4A). Furthermore, when nigericin is added on top of valinomycin/K^+^ at the steady state of Na^+^ uptake, Na^+^ exits the proteoliposomes very fast because of the collapse of both ΔpH and ΔΨ (Fig. 4A, filled inverted triangle). In contrast, when nigericin is added to the reaction mixture with no valinomycin/K^+^, Na^+^ exits much slower (Fig. 4A, empty inverted triangle). The slow exit rate of the latter may also be ascribed to the effect of the membrane potential produced by the electrogenic WT antiporter slowing the efflux rate.

To study the response of A167P to ΔΨ the mutant protein was purified and reconstituted into proteoliposomes and its ΔpH-driven ^22^Na^+^uptake activity was measured in the presence and absence of valinomycin/K^+^. Surprisingly, the permeant cation (valinomycin/K^+^) increased only slightly the rate of the uptake (Fig. 4B, filled squares). When the steady state of Na^+^ uptake was reached, addition of nigericin caused efflux at a rate much slower than that observed with the WT (Fig. 4B, filled inverted triangles compare to Fig. 4A). Taken together, the results indicate that the mutation either reduced the electrogenicity (H^+^/Na^+^ stoichiometry) and/or reduced the turnover of NhaA and/or changed a rate limiting step in the exchange mechanism of mutant A167P.

### H^+^/Na^+^ Stoichiometry of A167P-NhaA

As shown above (Fig. 4A), the activity of an electrogenic antiporter involves the electrophoretic movement of permeant ions, to compensate for charge translocation. The ratio between the movement of the counter ion and the rate of the antiporter is therefore a measure of the number of net charges transferred in one catalytic cycle. In the experimental setup described above (Fig. 4A) when ΔpH (acid inside) driven ^22^Na^+^ uptake was measured the initial rate of Na^+^ uptake was stimulated (4–5 fold) upon addition of 10 mM KCl (in the presence of 1 μM valinomycin). A similar effect was observed when RbCl wa added instead of KCl. Under these conditions (without K^+^), a ^86^Rb uptake was observed (Table 2). This uptake was dependent on the presence of valinomycin (not shown) and it was not observed when the Na^+^ concentration was far below the apparent *K*_m_ of the antiporter (data not shown), implying that the Rb^+^ uptake was dependent on the activity of the antiporter. The ratio of the fluxes of Na^+^/Rb^+^ of the WT was found to be very close to 1 (Table 2). This is the predicted ratio if 1 net charge is translocated per 1 Na^+^, as expected from the 2H^+^/1Na^+^ stoichiometery of WT NhaA. The ratio of the Na^+^/Rb^+^ fluxes of mutant A167P was around 1.3 (Table 2). This ratio clearly demonstrates that variant A167P is electrogenic with a H^+^/Na^+^ stoichiometry slightly higher than that of the WT.

**Table 2:** Na^+^/Rb^+^ uptake ratio indicates A167P electrogenicity.

	WT	A167P
	0.25 mM NaCl	
	μmol/min/mg protein	
^22^Na uptake	39.5±1.7	27.6±2.1
^86^Rb uptake	42±2.0	21.0±5.0
Na^+^/Rb^+^ ratio	0.9±0.1	1.3±0.3

Initial rates of ΔpH-driven ^22^Na and ^86^Rb uptake were measured for 5–60 s with or without valinmomycin, three to five repeats in each experiment. The uptake values and their ratio are shown with their standard error.

### Electrophysiology

**Figure 5:**
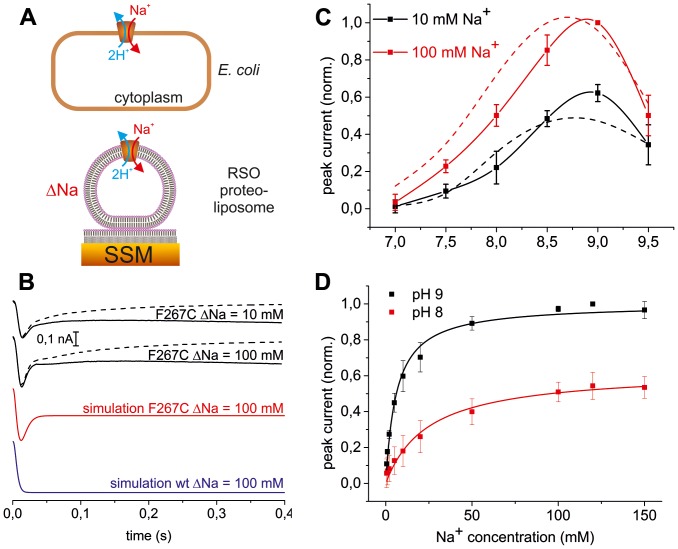
SSM-based electrophysiology of F267C NhaA. A) SSM-based electrophysiology. Upper panel schematically shows the right side out (RSO) orientation of the transporter in an *E coli* cell and the reverse activity mode of NhaA corresponding to the electrophysiological measurements. Lower panel shows a proteoliposome with right-side-out orientated NhaA adsorbed to the SSM. Transport is initiated by a Na^+^ concentration jump (ΔNa). Negative transient currents are detected by capacitive coupling via the liposome/SSM contact region. B) Currents recorded with proteoliposomes reconstituted with F267C NhaA after a 10 and 100 mM Na^+^ concentration jump (ΔNa) at pH 9.0. Other conditions as described in the text. The traces are recorded capacitive currents (black dashed line) and transporter currents reconstructed according to [31] (black solid line). The simulations were calculated as described in Materials and Methods based on the kinetic model shown in Fig. 6 using the parameters pK = 8.8, *K*_D_ for Na = 3 mM, k_1_ = 1000 s^−1^, k_2_ = 7000 s^−1^ (WT, blue line) and k_1_ = 1000 s^−1^, k_2_ = 50 s^−1^ (F267C, red line). C) pH dependence of the peak currents of mutant F267C recorded after Na^+^ concentration jumps of 10 and 100 mM. The peak currents were measured, corrected and normalized as given in Materials and Methods. Results shown are the average of three individual experiments ± SD. For comparison the pH profile of WT NhaA is included as a dashed line. D) Na^+^ dependence of the peak currents at pH 8 and 9. Other conditions and procedures are as in panel B. The solid lines are hyperbolic fits to the data yielding the *K*_M_ values for Na given in Table 3.

We have previously shown that NhaA mutant F267C, in helix IX, which is localized 18 Å apart from A167P, also lacks sensitivity to a change in ΔΨ [24]. Therefore, for a detailed study of their kinetics, both mutants, A167P [16] and F267C (Fig. 5), were subjected to SSM-based electrophysiology.

Na^+^ concentration jumps performed using F267C-NhaA proteoliposomes (Fig. 5A) gave rise to transient currents of negative polarity (Fig. 5B), which corresponds to a displacement of positive charge out of the liposomes. Consequently, as in the case of the A167P mutant [16], this showed that the F267C mutant is also electrogenic. A feature shared by both the A167P and the F267C mutants is their low turnover. The measured maximal peak currents in the mutants were more than 10 times lower than in the WT (Table 3).

**Table 3:** Kinetic parameters of the voltage insensitive NhaA mutants in comparison with the WT.

	I_max_ (pA)	*K*_m_ for Na (mM)	*K*_m_ for Na (mM)	pH_opt_	k_2_/k_1_
WT	−8000	11±1 (pH 8.5)	102±7 (pH 7.5)	8.7	7
A167P	−500	46±6 (pH 8.5)	285±14 (pH 7.5)	8.6	0.1
F267C	−500	6.8±2.4 (pH 9.0)	39±8 (pH 8)	8.9	0.05*

I_max_ = average values for transient currents at pH_opt_ and saturating Na concentration, pH_opt_ = pH of largest current at 100 mM Na^+^, k_2_/k_1_ =  ratio of rate constants of H^+^ translocation (k_2_) to Na^+^ translocation (k_1_). Values for WT and A167C from [15] and [16], values for F267C from electrophysiological experiments described in this report. (*) estimated by adjustment of simulation to data in Fig. 5B.

Currents recorded by SSM-based electrophysiology are transient (dashed lines in Fig. 5B) because they are detected by capacitive coupling via the proteoliposome/SSM contact region. However, using the known electrical properties of the proteoliposome/SSM compound membrane the transport currents generated by the Na^+^/H^+^ exchanger can be reconstructed [31] and are given as solid lines in Fig. 5B.

For the F267C mutant, currents were recorded following concentration jumps of 10 mM and 100 mM Na^+^ in the pH range of 7–9.5. The pH dependence of the peak currents (Fig. 5C) of F267C resembles that of WT NhaA [15]. The peak currents increase from almost 0 at pH 7 to a maximum which is reached at pH 9, and subsequently decrease at pH 9.5. Additionally, the Na^+^ dependence of the peak currents was recorded for the F267C mutant at two different pH values, 8 and 9 (Fig. 5D). From these recordings, we could determine *K*_m_ for Na^+^ of 6.8±2.4 mM at pH 9 and of 38.9±8.1 mM at pH 8 (Table 3). Similar values were reported for the WT, *K*_m_ for Na^+^ of 7.3±1.1 mM at pH 9 (see Table 3 and [15]). Remarkably, at pH values of 8.5 and above, mutant F267C produced biphasic transient currents which show a distinct negative pre steady-state current component (Fig. 5B). A similar behaviour at alkaline pH has previusly been observed with G338S NhaA and was assigned to Na^+^ translocation [15].

### Kinetic Parameters Determined from Dequenching and Electrophysiological Assays are Different

A comparison of Tables 1 and 3 reveals that in general the effect of the point mutation in positions 167 and 267 follows the same tendency namely decreasing cation affinity and turnover. However, the absolute values and the extent of the modification are significantly different for the two assay techniques. In addition, compared to WT NhaA, the pH profile of A167P NhaA is alkaline shifted by 1 pH unit (Fig. 3) while the pH optimum in the electrophysiological measurements is virtually unaltered in the variant [16] and the pK determined from the kinetic analysis of A167P NhaA increases by only 0.2 pH units (Table 3). A similar discrepancy was observed between ^22^Na uptake and SSM measurements [3]. Possible factors for these differences may be: i) The SSM measures current changes within milliseconds, whereas, the biochemical assays take seconds. ii) The natural environment in the membrane fragments compared to the more artificial environment in the reconstituted proteoliposomes used in electrophysiological measurements. As yet, this problem is unsolved and is under investigation. However, since the effect of the mutations observed by both techniques is very similar the discrepancy does not affect the conclusions drawn in this study.

## Discussion

We present a simple method to isolate randomly obtained mutants of NhaA impaired in either the translocation step and/or its unique pH response and describe in detail the selection of mutant A167P in proximity to the active site in TM V. Remarkably, the growth and biochemical characteristics of A167P were found very similar to those of F267C, a previously isolated mutant obtained by site directed mutagenesis [24]. Importantly, these mutants represent a novel class of NhaA mutants; whereas the WT is highly sensitive to a change in membrane potential due to its electrogenicity (a stoichiometry of 2H^+^/1Na^+^), these mutants are almost indifferent to such a change although they are also electrogenic. The reasons for this surprising phenomenon and its implications are explored here.

The selection method is based on the growth phenotype of the mutants in either *E. coli* EP432 or *E. coli* KNabc on selective media. Both *E. coli* strains EP432 [27] and KNabc [32] lack the Na^+^/Li^+^-specific antiporters NhaA and NhaB and the strain KNabc also lacks ChaA, the cation non-specific antiporter [26]. These strains cannot grow on the selective media (0.1 M LiCl at either pH 7 or pH 8.2 or 0.6 M NaCl at both pHs) without bearing a functional NhaA [5]. Therefore, their growth phenotype on only certain of the selective media suggest impairment in different properties of NhaA as described here through the selection steps of A167P. The selection of mutant A167P was conducted in *E. coli* KNabc but then it was transformed into *E. coli* EP432 and its growth phenotype was determined in both host strains (Table 1). In both hosts, an A167P expressing plasmid conferred an identical aberrant phenotype; As opposed to the WT which allows growth on all the selective media, *E. coli* EP432/A167P and *E. coli* KNabc/A167P grew on the non-selective medium (LBK) and only on the Li^+^ selective medium at neutral pH (Fig. 1B and Table 1). The identical phenotype conferred by A167P in both host strains implies that the mutation is not host specific. The amount of the mutant protein in the membranes of *E. coli* EP432 was 70% of the WT (Fig. 1C, Table 1). Similar to mutant A167P, mutant F267C was expressed in *E. coli* EP432 cells (95% of the WT) and grew on a selective medium only at neutral pH but it endured Na^+^ rather than Li^+^ (Table 1 and [24]).

The fact that both variants differed in the capacity to grow on the cations at neutral pH had first suggested that they were impaired in cation selectivity. However, this possibility was excluded because membrane vesicles isolated from *E. coli* EP432 cells expressing A167P and F267C showed Na^+^/H^+^ antiport activity ([24], Figs. 2 and 3) and Li^+^/H^+^ antiporter activity with alkaline shifted pH profile (Figs. 2 and 3). Furthermore, in proteoliposomes containing solely the A167P or F267C proteins Na^+^/H^+^ antiporter activity was detected (Fig. 4 and [24]).

Why can mutant A167P grow only on Li^+^ but not on Na^+^ selective medium at neutral pH? Various factors potentially contribute to the lack of growth of a mutant on the cation selective media at neutral pH. With respect to A167P, these can be: high *K*_m_ for Na^+^ compared to Li^+^ (Tables. 1 and 3), low turnover (Tables. 1 and 3) and more drastic alkaline shift of the pH profile (Fig. 3). Indeed, the apparent *K*_m_ for Na^+^ of variant F267C is lower than that of A167P and it grows on Na^+^ at neutral pH (Table 1). The growth on Li^+^ selective medium at neutral pH of F267C has not been determined.

Why are both strains unable to grow at alkaline pH in either the Na^+^ or Li^+^ selective media although both have an active antiporter at alkaline pH (Table 1, Figs. 2 and 3 and [24])? The toxicity of both Li^+^and Na^+^ drastically increase with pH [5] and both strains have an apparent *K*_m_, higher than the WT (Table 1) implying that in both strains the cation concentration increases in the cytoplasm and may reach the toxic level at alkaline pH. However, at least with Na^+^ we have previously observed growth at alkaline pH with such parameters of NhaA [33]. Therefore, the variants, most likely, are missing another factor which is an absolute requirement for growth at alkaline pH. High electrogenicity (a stoichiometry of 2H^+^/1Na^+^ ) [9] of the antiporter is an absolute requirement for growth of the cells at alkaline pH [5] because it allows the use of the membrane potential, the only driving force existing in *E. coli* at alkaline pH [34]. Indeed, the results summarized in Fig. 4 show that WT NhaA when reconstituted into sealed proteoliposomes without permeant ion exhibits a low rate and low steady state of ΔpH-driven Na^+^/H^+^ exchange activity because its electrogenic translocation creates a membrane potential that slows down its activity. Addition of a permeant ion (K^+^, valinomycin) collapses the membrane potential and both the rate and steady state of the Na^+^/H^+^ antiport increase by at least 4 folds. Therefore, it was very surprising to find out that collapsing the membrane potential across the A167P (Fig. 4) and F267C [24] proteoliposome membrane had very little effect on the mutant activity (at most the rate increases two folds). We therefore first assumed that these variants represent non electrogenic antiporters. However, this option was also ruled out for the following reasons: i) Electrophysiological measurements revealed that both A167P and F267C are electrogenic. Both generate transient currents in SSM-based electrophysiology (Fig. 5 and [16]). ii) The H^+^/Na^+^ exchange stoichiometry of A167P is close to∼ 2 H^+^ per Na^+^ (Table 2). In summary, although being electrogenic, the mutants do not react to membrane potential and therefore do not grow at alkaline pH.

**Figure 6:**
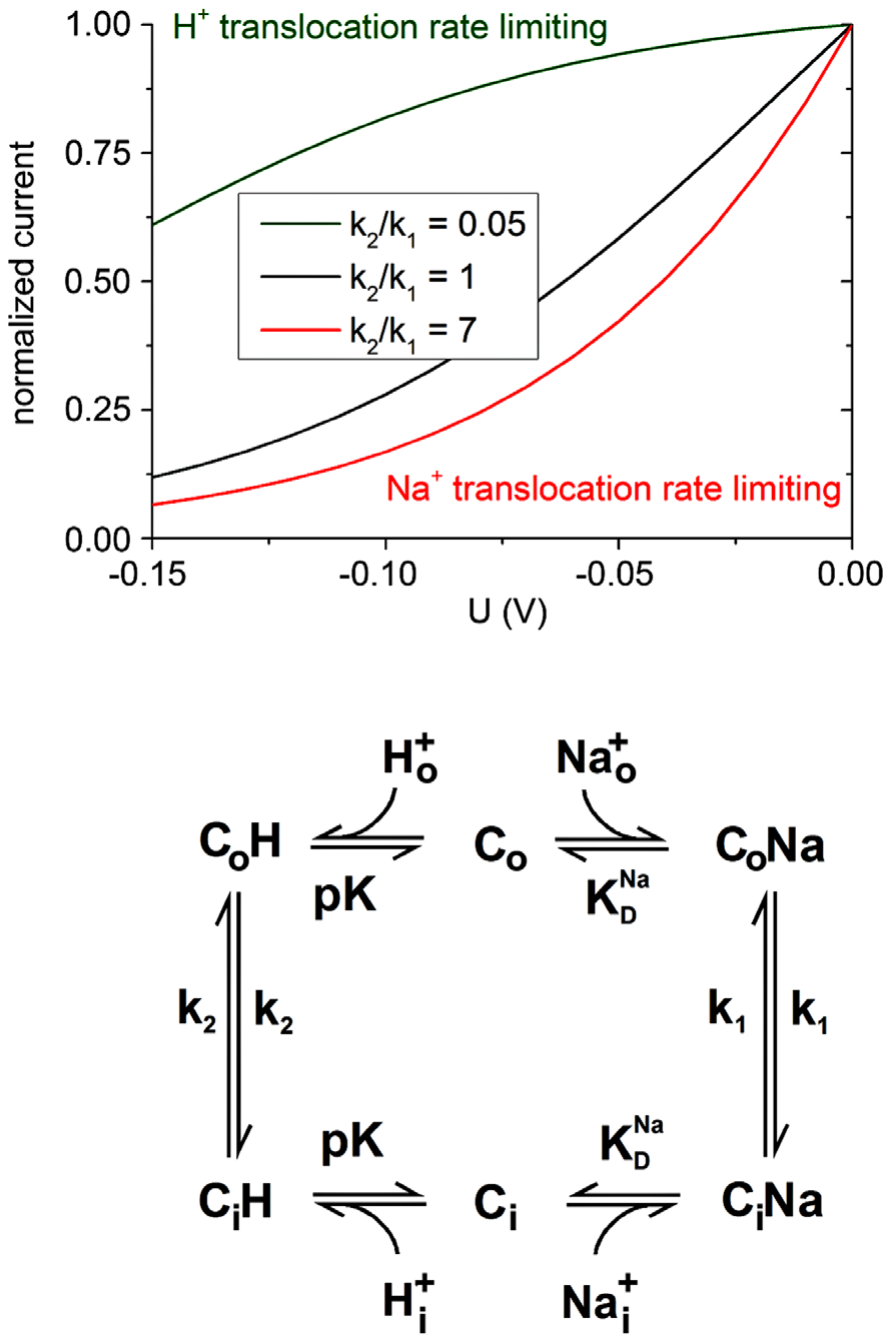
Current voltage relationship and kinetic model. Using the kinetic model given in the lower panel and the parameters for substrate binding of wild type NhaA (pK = 8.8, *K*_D_ for Na = 3 mM [15]) the current voltage relationship of the Na^+^/H^+^ exchanger was calculated. For comparison three different values of the k_2_/k_1_ ratio, the ratio of the rate constants of the Na^+^ and the H^+^ translocation reaction, were used in the calculation: k_2_/k_1_ = 7 corresponds to WT NhaA where Na^+^ transport is rate limiting. k_2_/k_1_ = 0.05 corresponds to F267C NhaA where H^+^ transport is rate limiting. k_2_/k_1_ = 1 is an intermediate case. For comparison, currents are normalized to their respective values at voltage U = 0V.

How can an electrogenic transporter not react to a change in the membrane potential? Based on our electrophysiological analysis [15] we have recently suggested a mechanistic model for the electrogenic behavior of WT NhaA taking into account that two negatively charged residues, D163 and D164, constitute the Na^+^ binding site ([25] and Fig. 6). Therefore, Na^+^ translocation, i.e. the conformational transition of the Na^+^ loaded carrier C_i_Na→C_o_Na, (with rate constant *k*_1_ in the kinetic model Fig. 6) is associated with the displacement of 2 negatively charged aspartate residues plus the Na^+^ ion resulting in the displacement of one net negative charge. Indeed Na^+^ translocation was experimentally verified to generate a negative charge displacement [15]. In contrast, during H^+^ translocation C_o_H→C_i_H (with rate constant *k*_2_) the two H^+^ ions fully compensate the two negative aspartate charges leading to an electroneutral reaction. Since in WT NhaA, electrogenic Na^+^ translocation is rate limiting [15], turnover is strongly voltage dependent in the WT.

Now we propose that both A167P and F267C mutations selectively slow down H^+^ translocation to an extent that this step becomes rate limiting, rendering the rates of carriers independent of membrane potential as experimentally observed (Fig. 4). Indeed, both A167P NhaA [16] and F267C NhaA (Fig. 5C) show a stronger down regulation in the alkaline range compared to WT NhaA implying rate limitation by the protonation induced partial reaction.

Crucial support for this interpretation comes from kinetic analysis (Fig. 5B-D). The sodium dependence at pH 7.5 and 8.5, as well as the pH dependence at 10 and 100 mM Na^+^ of A167P NhaA can be simultaneously fitted with a *k*_2_/*K*_1_ ratio of 0.1 [16] i.e. Na^+^ translocation in the mutant is much faster than H^+^ translocation. Analysis of the shape of the current transients of F267C NhaA further corroborated this contention. In contrast to WT, a pre-steady-state component was observed in the recorded transient currents (Fig. 5B). This signal is similar to that obtained with NhaA mutant, G338S at very alkaline pH, when H^+^ translocation is drastically slowed down [15] and may therefore be taken as evidence for slow H^+^ translocation. Indeed, we calculate a signal for F267C (Fig. 5B, red line) very similar to the experimentally observed current using a 20 times slower H^+^ than Na^+^ translocation rate (*k*_2_/*K*_1_ = 0.05) In contrast, the WT *k*_2_/*K*_1_ of 7 yields a monophasic negative current (Fig. 5B, blue line).

In conclusion, for A167P as well as for F267C NhaA it seems clear that H^+^ translocation is rate limiting. Incidentally, a drastically reduced H^+^ translocation rate also accounts for the 10 - 20 times lower turnover of the mutants observed in the electrophysiological experiments (Table 3). Note that low turnover could contribute to the apparent indifference to a change in membrane potential of Na^+^ uptake of the mutants, because low turnover results in a lower membrane potential. In any case, our electrophysiological study reveals that a switch of the rate limiting step from an electrogenic to an electroneutral reaction in the reaction cycle of an electrogenic secondary transporter can yield a phenotype of an electroneutral transporter.

Model based calculation of the effect of membrane potential on an electrogenic Na^+^/H^+^ exchanger is shown in Fig. 6. The pH and Na^+^ dependence of F267C NhaA as revealed by electrophysiology, was found to be very similar to the WT. We can, therefore, calculate the behaviour of the transporter under arbitrary conditions such as in the presence of a membrane potential using wild type parameters and a modified *k*_2_/*K*_1_ ratio. Figure 6 shows Na^+^/H^+^ exchanger activity driven by a Na^+^ gradient (high Na^+^ outside the vesicles, the reverse of the physiological situation) as is the case for our electrophysiological [15] as well as the ^22^Na^+^ uptake measurements [8]. The calculation was performed for different values of *k*_2_/*K*_1._ A *k*_2_/*K*_1_ value of 7 corresponds to the wild type situation, where Na^+^ translocation is rate limiting, *k*_2_/*K*_1_ value of 0.05 corresponds to F267C NhaA with rate limitation by H^+^ translocation and *k*_2_/*K*_1_ = 1 is an intermediate case for comparison. It is clear from the figure that WT turnover decreases rapidly with increasing negative potential while F267C (*k*_2_/*K*_1_ = 0.05) retains most of its activity even at a Potential U = −150 mV. These simulations support our claim that in A167P and F267C NhaA a switch of rate limitation from the Na^+^ to the H^+^ translocation step leads to a phenotype that is insensitive to the membrane potential.

**Figure 7:**
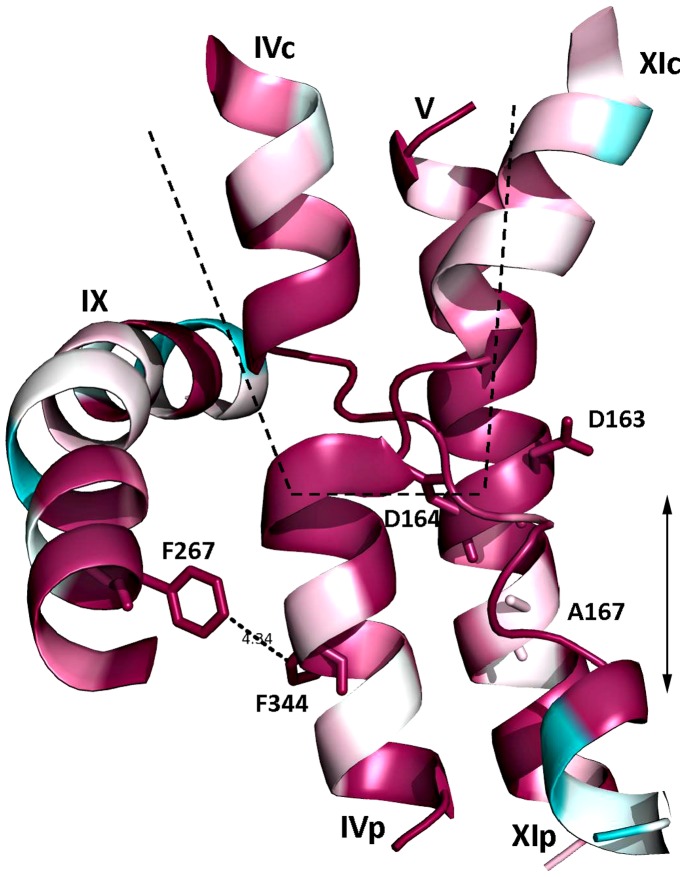
Residues F267 and A167 are located in proximity to the TMs IV/XI assembly of NhaA. In silico ConSurf analysis (http:/conserve.tau.ac.il) was conducted on the TMs IV/XI assembly with the extended chains in the middle, TM V with the binding site (Asp163 and Asp164) and Ala167 and TM IX with Phe267. A ribbon representation is shown using the color cod, turquoise to maroon indicating variable to conserved residues respectively. The picture was generated using MOLSCRIPT and Raster 3D. The cytoplasmic funnel and the barrier are marked (dashed line and double head arrow respectively).

Insights from the crystal structure (20) are summarized in Fig. 7. The amino acids Ala167 and Phe267 are highly conserved residues ([35] and Figure 7), implying an important structural/functional role in NhaA. Accordingly, both mutants have similar kinetic properties and a similar phenotype of growth (Table 1). However, compared to WT, they have a drastically different ratio k_2_/k_1_ (Table 3). Why are these variants, which are 18 Å apart, so similar in properties? The NhaA crystal structure [19] solves the puzzle. The positions of both Phe267 and Ala167 are strategic: Phe267 in TM IX is in direct contact with Phe344 of TM IVp (about 4 Å, Fig. 7 and [19]). TM IV is part of the TMs IV/XI assembly which contains the interrupted helices in proximity to the binding site [19]. The connection between Phe267 and the assembly (Phe344) was found critical for NhaA activity; whereas, single Cys replacement of each residue was active, the double Cys replacement (F267C/P344C) was lethal [24]. The position of Ala167 is next to the binding site (D163, D164) and the extended chains of the TMs IV/XI assembly. We have recently shown that this extended chain changes conformation with pH [36]. Finally, both Ala167 and Phe267 are located within the periplasmic barrier which separates the cytoplasmic funnel from the periplasmic funnel of NhaA ([19] and Fig. 7). This barrier as observed at pH 4 does not allow any ion to cross the antiporter. It is therefore understandable that replacements of strategic residues in this barrier would change the cation exchange activity of NhaA.

## Materials and Methods

### Plasmids, Bacterial Strains and Culture Conditions

Plasmid pAXH3 is a pET20b (Novagen) derivative. It encodes a His-tagged NhaA [37], lacks the BglII site at position 3382 and containg a BstXI silent site at position 248 in *nha*A. The NhaA protein mutants were named by the mutational change. For example, the plasmid and protein bearing the Pro replacement mutation at Ala167 (A167P) were named, pAXH3-A167P. pAXH3-F267C was isolated previously [24].

EP432 is an *Escherichia coli* K-12 derivative, which is *mel*BLid, Δ*nha*A1::*kan*, Δ*nha*B1::*cat*, Δ*lac*ZY, *thr*1 [27].

TA16 is *nha*A^+^ *nha*B^+^ *lac*I^Q^ and otherwise isogenic to EP432 [8]. KNabc is an *E. coli* mutant derived from TG1 of which major Na^+^/H^+^ antiporters were inactivated (NhaA^−^, NhaB^−^, ChaA^−^) [32] [38].

Cells were grown in either L broth (LB) or modified L broth (LBK, with NaCl replaced by KCl) [39]. The medium was buffered with 60 mM 1,3 bis-{tris (hydroxymethyl)-methylamino} propane (BTP). For plates, 1.5% agar was used. For induction, the cells were also grown in minimal medium A [40] without sodium citrate and with 0.5% (w/v) glycerol, 0.01% (w/v) MgSO_4_·7H_2_O and 2.5 μg/mL thiamine and threonine when needed. Antibiotics were 100 μg/mL ampicillin and/or 50 μg/mL kanamycin. To test resistance to Li^+^ and Na^+^, EP432 cells transformed with the respective plasmids were grown on LBK to O.D._600_ of 0.6–0.7. Samples (4 μL) of serial 10-fold dilutions of the cultures were spotted onto agar plates containing the indicated concentrations of NaCl or LiCl at the various pHs and incubated for 24 h or 48 h at 37°C.

### Mutagenesis

For random mutagenesis we used a PCR-based protocol with the kit GeneMorph II (Stratagene, USA) and pAXH3 as a template. The mutagenized plasmids were transformed into KNabc cells and plated on LBK plates and then each colony was replica plated on the selective medium plates. Plasmids from colonies with aberrant phenotype were isolated, retransformed to KNabc and re-plated on selective plates to verify that the phenotype is encoded by the plasmid and not by the genome. Finally, the *nha*A gene DNA was sequenced to identify the mutation. The mutation bearing plasmid was re-transformed into EP432 cells and re-plated on LBK plates and then on the selective plates to ensure that the mutation is independent of the host stain.

### Isolation of Everted Membrane Vesicles and Measurement of Na^+^/H^+^ Antiporter Activity

Everted membrane vesicles from EP432 or KNabc cells transformed with the respective plasmids were prepared as previously described [41], [42] and used to determine Na^+^/H^+^ or Li^+^/H^+^ antiporter activity. The antiporter activity assay was based on the measurement of Na^+^- or Li^+^-induced changes in the ΔpH as measured by acridine orange, a fluorescent probe of ΔpH. The fluorescence assay was performed in a 2.5 mL reaction mixture containing 100–150 μg membrane protein, 0.1 μM acridine orange, 150 mM choline chloride, 50 mM BTP and 5 mM MgCl_2_, and pH was titrated with HCl. After energization with D-lactate (2 mM), fluorescence was quenched (Fig. 2A, downward pointed arrow) and achieved a steady state, and then 10 mM of either Na^+^ or Li^+^ was added (upward pointed arrow). A reversal of the fluorescence level (dequenching) indicates that protons are exiting the vesicles in antiport with either Na^+^ or Li^+^. As shown previously, the end level of dequenching is a good estimate of antiporter activity, and the ion concentration that gives half-maximal dequenching is a good estimate of the apparent *K*_m_ of the antiporter activity [43], [44]. The concentration range of the tested cations was 0.01 to 100 mM at the indicated pHs and the apparent *K*_m_ values were calculated by linear regression of a Lineweaver-Burk plot.

### Overexpression and Purification of the NhaA Mutant Variants

Overexpression of the NhaA mutants and affinity purification (Ni^+^-nitrilotriacetic acid-agarose, Qiagen) were performed as described previously [24], but the protein was eluted in a buffer containing 300 mM imidazole, 25 mM citric acid, 100 mM choline chloride, 5 mM MgCl_2_, 10% glycerol and 0.015% n-dodecyl-D-maltopyranoside (DDM) (final pH was 4). Sucrose (10%) was added to the eluted protein solution and the mixture was dialyzed overnight at 4°C in the acidic elution buffer containing 10% sucrose. The affinity purified protein was frozen in liquid nitrogen and stored at 80°C.

### Reconstitution of NhaA Variants into Proteoliposomes and Measurement of ΔpH-Driven ^22^Na Uptake and Na^+^/H^+^ Stoichiometry

NhaA proteoliposomes were reconstituted and ΔpH (acidic inside)-driven ^22^Na uptake was determined as described previously [8], [28]. ^86^Rb uptake experiments were done practically in the same way except that valinomycin (1 μM) and 1 μCi ^86^RbCl (1 mM) were added instead of ^22^NaCl [45]. All experiments were done in duplicates and repeated at least twice with practically identical results.

### Detection and Quantitation of NhaA and its Mutated Derivatives in the Membrane

Total membrane protein was determined according to Bradford [46]. The expression level of His-tagged NhaA mutants was determined by resolving the Ni-NTA-purified proteins by SDS-PAGE, staining the gels with Coomassie blue and quantifying the band densities by Image Gauge (Fuji) software [37].

### SSM-based Electrophysiology

SSM measurements were performed essentially as previously described [15]. In brief, 30 μL of proteoliposomes containing F267C-NhaA at a lipid concentration of 5 mg/mL (lipid/protein ratio, LPR = 10) were allowed to adsorb to an octadecanethiol/phospholipid hybrid bilayer on a gold surface (sensor) for 2–3 h. A single solution exchange protocol was used to initiate electrogenic transport (nonactivating solution 0.5 s; activating solution 0.5 s; nonactivating solution 0.5 s). Currents were amplified using a current amplifier set to a gain of 10^9^ V/A and a rise time of 10 ms. Both nonactivating and activating solutions contained 25 mM Tris, 25 mM MOPS, 25 mM MES, 5 mM MgCl_2_ and 1 mM dithiothreitol and were adjusted to the desired pH using HCl or Tris. Nonactivating solutions additionally contained 300 mM KCl, while activating solutions contained instead *x* mM NaCl and (300– *x*) mM KCl. Peak currents were corrected for solution exchange effects caused by the unspecific effect of Na^+^ concentration jumps on the SSM by subtracting the peak currents recorded for Na^+^ concentration jumps at pH 6, where no F267C-NhaA-specific currents of negative polarity were detected.

### Simulation of the Transient Currents

Based on a kinetic model presented previously (Fig. 6) a pre steady-state solution was determined. The numerical solution was calculated by solving the defining differential equations of the kinetic model using Berkeley Madonna (version 8.3.18; Berkeley Madonna Inc., University of California, Berkeley, CA, USA). To account for the limited speed of the solution exchange at the sensor surface the calculated current was subsequently numerically filtered using a 3rd order low-pass filter. The time constant of the filter (τ = 4.5 ms) was chosen to best fit the transient currents generated by the Na^+^ concentration ejumps. For the model calculation shown in Fig. 6 the steady-state solution was calculated as described in [15].
