# Correction: NhaA Na^+^/H^+^ Antiporter Mutants That Hardly React to the Membrane Potential

**DOI:** 10.1371/journal.pone.0102206

**Published:** 2014-07-17

**Authors:** The PLOS ONE Staff

The third author's name is spelled incorrectly. The correct name is: Maral Budak. The correct citation is: Alkoby D, Rimon A, Budak M, Patino-Ruiz M, Călinescu O, et al. (2014) NhaA Na^+^/H^+^ Antiporter Mutants That Hardly React to the Membrane Potential. PLoS ONE 9(4): e93200. doi:10.1371/journal.pone.0093200
